# Editorial: Bridging the initiation of a response hypothesis in autism: from language to motor action

**DOI:** 10.3389/fpsyg.2023.1281336

**Published:** 2023-09-25

**Authors:** Joana C. Carmo, Alan Langus, Carlos N. Filipe

**Affiliations:** ^1^Digital Human-Environment Interaction Labs (HEI-Lab), Lusófona University, Lisboa, Portugal; ^2^Department of Linguistics, University of Potsdam, Potsdam, Germany; ^3^NOVA Medical School, Universidade Nova de Lisboa, Lisboa, Portugal

**Keywords:** autism spectrum disorder, response initiation, apathy, avolition, executive functions

Verbal fluency tasks are a classical neuropsychological tool and have been widely used for assessing executive functioning but also semantic integrity. Switching and Clustering measures were proposed as qualitative indicators of the two systems, respectively (Troyer et al., 1997). In autism, a very characteristic pattern was found, with a discrepancy between an impoverish overall performance, that could not be explained by difficulties either in Clustering or Switching (Spek et al., 2009; Inokuchi and Kamio, 2013). This pattern led Spek et al. (2009) to raise the possibility that the explanation for it could reside in an isolated initiation deficit in autism spectrum disorder (ASD) (see also Reverberi et al., 2006 for a similar reasoning).

Trailing on this idea, Carmo et al. (2015) designed a verbal fluency study aimed at directly evaluating this hypothesis. The characteristic pattern of performance in autism was once again found, but critically, the authors showed that the impoverish performance of autistic participants was only observed in the first time-bin analyzed, while in the remaining of the test, the performance was intact (Carmo et al., 2015, 2017). This finding led the authors to classify these results as preliminary evidence supporting a response initiation impairment hypothesis in autism (Carmo et al., 2015). In a follow up study, Carmo et al. (2017) have not only replicated their previous findings but showed how to circumvent the response initiation difficulties felt by individuals on the spectrum, by prompting them with simple external verbal cues.

From two qualitative studies of first-hand accounts, additional support can be found for these ideas (Welch et al., 2019; Buckle et al., 2021). Note, for instance, that in the work of Buckle et al. (2021) on inertia, two main and consistent themes emerged: the difficulty in initiating even simple actions and that external prompting promotes and supports actions. Despite the fact that a dysfunction of the executive system in autism is one of the most prominent accounts of the condition (e.g., Russel, 1997), and that this hypothesis has been tested for decades now in all sorts of behavioral tasks, yet the initiation of a response (Shallice and Burgess, 1991), a highly pervasive executive function, has been oddly neglected. In the words of the authors in this volume, this cognitive domain was considered as “likely the less studied executive domain” (Carmo and Filipe, p. 3) or suffering from “under-recognition” (Buckle et al., p. 2). In addition, a particular appeal of this hypothesis of troubles at initiating responses, resides in the fact that, as mentioned above, external prompting seems to ameliorate the difficulties felt by autistic individuals (Carmo et al., 2017; Buckle et al., 2021).

To add up, the corpus of knowledge on this topic might be scattered. A possible contribution for the dispersion of data in this field, is the vast and diverse terminology used by different parties and disciplines, a problem that was diagnosed by Buckle et al.. The lack of or limited response initiation overlaps somehow with many other terms and similar phenomena, as *apathy* or its severe cases—*catatonia* (Yon-Hernández et al.), *avolition* (in Buckle et al.) or *getting stuck* (Greaves-Lord et al.). In Buckle et al. a collection of many disjoint terminology was put together with their particular and differentiating meanings and contexts.

Importantly, two papers, in this dedicated issue, attempt at bridging the gap between scientific knowledge and practice. Yon-Hernández et al. address adaptive skills in daily activities, whereas Greaves-Lord et al. address interventive approaches directed at different primary stakeholders (e.g., parents, care-providers).

A decade-long work led Greaves-Lord et al. to describe the phenomenon of *state of stuckness*. This work was based mainly on qualitative interviews and focus groups with autistic people but also qualitative content from family members and a large panel of experts. They present a summary of main results from the *Grasp on Behavior* and *Dealing with (one's own) Autism* projects, describing several contributing factors to the limited societal participation by people on the spectrum (e.g., schooling drop-out) (Greaves-Lord et al.). An outline of different interventive approaches can be found in this work, having the ultimate aim of “moving away from this stuck state to a more flexible, limber supple states” (Greave-Lord et al., p. 1).

In the work of Yon-Hernández et al., it is explored how much IQ, executive functioning, or autistic symptomatology do predict adaptive skills in everyday tasks (they also included a group of patients with Schizophrenia). They used objective executive tasks as well as a subjective self-reporting questionnaire (DEX-Sp), which comprises a specific subscale on disorganization and apathy. Results show that, besides differences from control participants on shifting and on both subjective subscales, both objective and subjective executive measures are predicting the adaptive skills level (Yon-Hernández et al.), with the subjective ones being predictive to a greater extent. These findings are similar to what was observed by Yon-Hernández et al. (2022) on a previous study with a larger sample. Interestingly, it was the apathy subscale that led to greater difficulties at daily living skills, which could signify that the ability for planning and initiating appropriate behaviors might be particularly heavy on daily activities (Yon-Hernández et al., 2022; Yon-Hernández et al.).

Note that in that work, inhibitory processes in ASD were found to be intact, which brings us to the research conducted by Carmo and Filipe. When studying executive processes, or other cognitive domains, it is important to recognize that any experimental task is very unlikely a pure measure of a single cognitive domain. And that the pervasive nature of response initiation on all sort of behaviors might have contaminated a host of research on cognition in autism. Carmo and Filipe clearly show, that was the case when inspecting executive functions, namely inhibitory control. In their work, with adults on the spectrum, they demonstrated that the apparent deficits in inhibition were canceled out when correcting for the response initiation component, which they found, once again, deficient.

Buckle et al. offer a transdiagnostic perspective on different pathologies, such as Parkinson's disease, schizophrenia, and autism, where initiation impairments are evident. The authors censure the arbitrary distinctions between disciplines (e.g., neurology, psychiatry) and claim that those distinctions have an impact on how these deficits are explained, and that the similarities between different conditions remain unexplored (Buckle et al.). In all three conditions, there is for instance, an overlap in negative symptoms, and core to them is the ability to act on intentions (Buckle et al.). Critically, the authors propose in this work that a triad of interacting elements are called and in need for successfully initiating an action: (i) executive functioning; (ii) volition and (iii) movement. As a result, impairments on at least one of these elements would lead to a failure to initiate a response (Buckle et al.).

In addition to the vast amount of different terminology in use and the seclusion of knowledge from close by disciplines, the covert nature of initiation processes was mentioned as an added problem (see Buckle et al.). As such, failures at initiating a response can be mis-interpreted as behavioral *defiance* or *oppositional* (in Greaves-Lord et al.) or *non-compliance or withdrawal* (in Buckle et al.).

It is our opinion, that the hypothesis of initiation impairments in autism deserves further research efforts, that are solid and systematic, convergent and far-reaching.

## Author contributions

JC: Writing—original draft, Writing—review and editing. AL: Writing—review and editing. CF: Writing—review and editing.
